# Noncoding RNAs regulate alternative splicing in Cancer

**DOI:** 10.1186/s13046-020-01798-2

**Published:** 2021-01-06

**Authors:** Yunze Liu, Xin Liu, Changwei Lin, Xianhong Jia, Hongmei Zhu, Jun Song, Yi Zhang

**Affiliations:** 1 Department of General Surgery, Affiliated Hospital of Xuzhou Medical University, Xuzhou, 221000 China; 2 Department of Traditional Chinese Medicine, Affiliated Hospital of Xuzhou Medical University, Xuzhou, 221000 China; 3 Department of Endocrinology, Affiliated Hospital of Xuzhou Medical University, Xuzhou, 221000 China; 4 Department of Gastrointestinal Surgery, the Third XiangYa Hospital of Central South University, Changsha, 410013 China

**Keywords:** Alternative splicing, Cancer, Noncoding RNA, miRNA, lncRNA, circRNA, snRNA

## Abstract

AS (alternative splicing) is a fundamental process by which a gene can generate multiple distinct mRNA transcripts to increase protein diversity. Defects in AS influence the occurrence and development of many diseases, including cancers, and are frequently found to participate in various aspects of cancer biology, such as promoting invasion, metastasis, apoptosis resistance and drug resistance. NcRNAs (noncoding RNAs) are an abundant class of RNAs that do not encode proteins. NcRNAs include miRNAs (microRNAs), lncRNAs (long noncoding RNAs), circRNAs (circular RNAs) and snRNAs (small nuclear RNAs) and have been proven to act as regulatory molecules that mediate cancer processes through AS. NcRNAs can directly or indirectly influence a plethora of molecular targets to regulate cis-acting elements, trans-acting factors, or pre-mRNA transcription at multiple levels, affecting the AS process and generating alternatively spliced isoforms. Consequently, ncRNA-mediated AS outcomes affect multiple cellular signaling pathways that promote or suppress cancer progression. In this review, we summarize the current mechanisms by which ncRNAs regulate AS in cancers and discuss their potential clinical applications as biomarkers and therapeutic targets.

## Background

AS (alternative splicing) of RNA involves the way in which pre-mRNA (pre-messenger RNA) undergoes splicing at different splice sites, and introns are selectively removed while exons are retained to produce two or more kinds of mRNA splicing isoforms. AS progression has been discovered in over 95% of human multiexon genes and has profound consequences at the protein level, increasing the diversity of gene phenotypes and protein functions in eukaryotes [1, 2]. AS allows pointed splicing isoforms expressed at a given time to participate in various cellular activities, while AS defects are related to various diseases. In recent decades, it has been observed that many AS changes play vital roles in cancer progression. Cancer cells subvert normal AS events to produce special splicing isoforms that affect cellular functions and potentially control cell proliferation and tumorigenesis. Gaining an understanding of AS mechanisms may reveal new opportunities to develop targeted cancer treatment strategies [3–5].

AS is a highly controlled process, and with the use of high-throughput sequencing and RNA-seq technologies, thousands of ncRNAs (noncoding RNAs) have been identified crucial to regulate AS at multiple levels in cancers [6]. Here, we review our current knowledge of ncRNAs as AS regulators, their mechanisms of action in cancer, and their contributions to the diagnosis and treatment of cancer.

## Alternative splicing in cancer

**Fig. 1:**
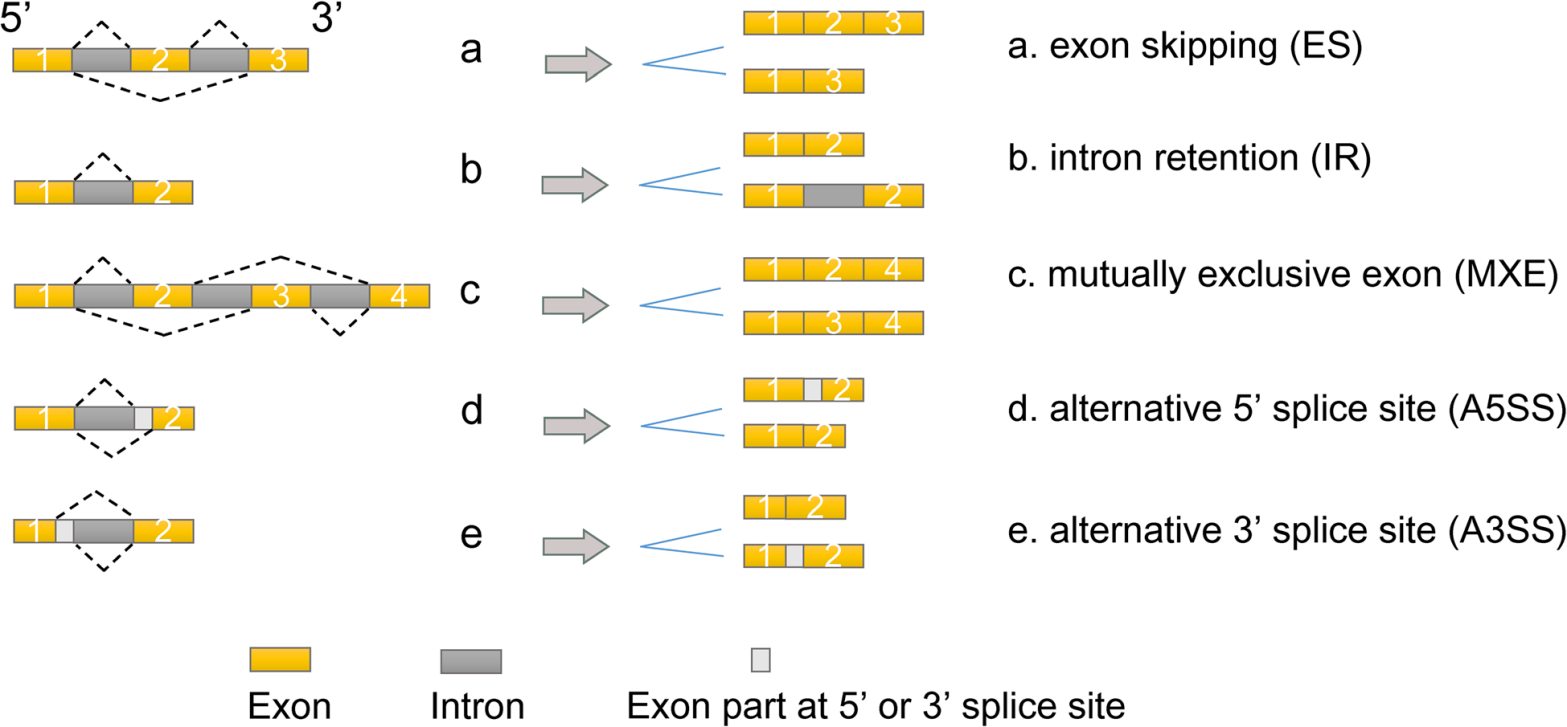
**Different types of AS. a**. ES (exon skipping); **b**. IR (intron retention); **c**. MXE (mutually exclusive exon); **d**. A5SS (alternative 5′ splice site); and **e**. A3SS (alternative 3′ splice site)

AS relies mainly on the spliceosome, splice site, cis-acting element and trans-acting factor. The spliceosome is assembled from five snRNPs (small nuclear ribonucleoproteins), namely, U1, U2, U4, U5, U6 and over 300 non-snRNP proteins. In AS, spliceosomes recognize splice sites located at the borders between introns and exons, including 5′ splice sites, 3′ splice sites and branch points of pre-mRNA. Through recognition, spliceosomes convene and function as ribozymes to catalyze the removal of introns [7]. Then, retained exons link together to eventually generate the mRNA, forming five different types of AS: ES (exon skipping), IR (intron retention), MXE (mutually exclusive exon), A5SS (alternative 5′ splice site) and A3SS (alternative 3′ splice site) (Fig. 1). During the AS process, the spliceosome cannot interact with the splice site independently to determine the gene sequences of the pre-mRNA that are retained or skipped. Cis-acting elements and trans-acting factors are needed in the recognition and selection of splice sites and in the aggregation of spliceosomes [8].

**Fig. 2:**
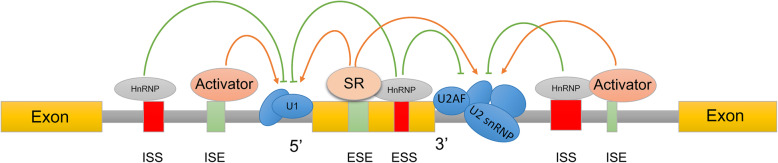
A schematic representation of AS regulated by cis-acting elements and trans-acting factors. The cis-acting elements ESEs and ESSs represent exonic splicing enhancers and silencers, respectively. ISEs and ISSs represent intronic splicing enhancers and silencers, respectively. SR proteins, hnRNPs, or other trans-acting factors bind to cis-acting elements for positive or negative AS regulation by affecting the recruitment or recognition of spliceosome components to splice sites; for example, U1 binds to the 5′ splice site, while U2AF and U2 bind to the 3′ splice site

Cis-acting elements, categorized as ISEs (intron splicing enhancers) and ISSs (silencers), as well as ESEs (exon splicing enhancers) and ESSs (silencers), are the only positions beside variable exons and side-by-side introns to provide gene sites of binding with trans-acting factors [9] (Fig. 2).

Trans-acting factors in AS are mainly SFs (splicing factors), which include three common types [10]: SR (serine/arginine-rich) proteins, hnRNPs (heterogeneous nuclear ribonucleoproteins) and tissue-specific SFs. SR proteins, composed of 12 members (serine/arginine-rich splicing factor 1–12, SRSF1–12), generally bind ESE sequences and promote the recognition of exons by recruiting U1 to the 5′ splice site and U2AF (U2 auxiliary factor) and U2 to the 3′ splice site [11, 12]. HnRNPs mainly bind ESS or ISS sequences and inhibit the recognition of exons by hindering contact between spliceosomes and splice sites [13]. Tissue-specific SFs, such as RBM5 (RNA binding motif protein 5), RBM24, QKI (quaking), and Nova1 (NOVA alternative splicing regulator 1), exist in special tissues and allow more specific controls of splice site selection for cell specification and organ function. For example, RBM24 binds an ISE to promote muscle-specific exon inclusion in cardiac and skeletal muscles [14]. In addition to the above three classical SFs, some other regulatory proteins, such as U2AF65, SF3B3 (splicing factor 3b subunit 3), and SF1, have also been classified as SFs by binding directly to pre-mRNAs.

In addition to cis-elements and trans-acting factors, as the excision of most introns occurs cotranscriptionally, AS is coupled with pre-mRNA transcription [15, 16]. Factors or chromatin modifications related to pre-mRNA transcription are able to affect AS. For instance, a fast rate of Pol II (RNA polymerase II) elongation, which contributes to transcription elongation, induces spliceosome aggregation at the stronger alternative 3′ splice site (A3SS), which is located downstream of the weaker A3SS, leading to exon skipping [10]. Moreover, the chromatin modification H3K36me3 (trimethylation on histone H3 thirty-sixth lysine), which can promote transcriptional activation, results in the recruitment of the SF SRSF1 to pre-mRNA to regulate AS [17].

In cancers, AS defects have been repeatedly observed and result from mutations in genes that encode cis-acting elements [18] or SFs [19] or from misregulations in the AS machinery [5, 20]. For example, in hematologic malignancies, a gene mutation in the SF SRSF2, which regulates the AS of the EZH2 pre-mRNA, triggers a reduction in EZH2 isoform expression [21]. In CRC (colorectal cancer) samples, overexpression of the SF SRSF6, which binds to the ZO-1 motif in exon 23, can contribute to the expression of an alternative isoform of ZO-1 due to exon 23 skipping [22].

The outcome of aberrant AS brings numerous special splicing isoforms at the protein level and gives rise to the initiation and progression of cancer. In PCa (prostate cancer), abnormal splicing of the androgen receptor pre-mRNA produces splicing isoforms that promote drug resistance and cancer aggression [23]. In human skin cancer, a splicing alteration in the protein TNC (tenascin C) pre-mRNA increases the number of tumor-promoting TNC-FL isoforms, resulting in cancer cell invasion and metastasis [24].

## Noncoding RNAs in alternative splicing

NcRNAs, which occupy almost 60% of the transcriptional output in human cells [25], are RNAs that do not encode proteins. With the development of sequencing technologies, a multitude of ncRNAs have been discovered and classified based on their size or function into many kinds, including miRNAs (microRNAs), siRNAs (small interfering RNAs), piRNAs (piwi-interacting RNAs), rRNAs (ribosomal RNAs), snRNAs (small nuclear RNAs), lncRNAs (long noncoding RNAs), or circRNAs (circular RNAs).

MiRNAs are small ncRNAs that are approximately 19–25 nucleotides in length with highly conserved properties [26]. MiRNA is commonly assembled into the RISC (RNA-induced silencing complex), which is guided to degrade mRNA or simply inhibit mRNA translation without changing mRNA levels, depending on the complete or partial complementation of miRNA. The miRNA-mediated action occurs mainly in the 3′ UTR (untranslated region) and sometimes in the 5’UTR of the target mRNA [27–30]. Moreover, some miRNAs are able to stabilize mRNAs or promote translation by binding to the mRNA 5’UTRs [31].

LncRNAs are more than 200 nucleotides in length with poorly conserved properties, and their functions include acting as molecular scaffolds in various biological processes, disturbing nearby pre-mRNA transcription, forming RNA–RNA duplexes with pre-mRNA molecules or miRNAs as posttranscriptional regulators, promoting specific protein activities, and recruiting or inhibiting regulatory proteins in multifarious transcriptional, posttranscriptional or posttranslational manners. Additionally, several lncRNAs encode peptides or generate small RNA molecules that exert biological effects [6, 32–34].

CircRNAs are closed endogenous ncRNAs that are more stable than linear RNAs due to their structures, which are covalently closed continuous loops without a 5′ cap structure and 3′ polyadenylated tail [35]. CircRNAs can sponge miRNAs through base pairing to protect their target mRNAs. Moreover, circRNAs are able to inhibit or recruit specific proteins to function in the regulation of various biological processes, to enhance particular protein functions to facilitate gene transcription, and to serve as protein scaffolds to promote the reaction of enzymes and substrates. Additionally, some circRNAs may have coding potential to produce peptides under certain conditions [36, 37].

SnRNAs (U1 ~ U7) average approximately 150 nucleotides in length with highly conserved properties and participate in various aspects of RNA biogenesis as core components of functional snRNPs [38, 39]. On the one hand, snRNAs associate with proteins to form snRNPs and play snRNP-mediated regulatory roles. On the other hand, snRNAs can form dynamic base pairing interactions between snRNAs and between snRNAs and the pre-mRNA, ensuring the coordination of pre-mRNA processing [38, 40].

According to current studies, ncRNAs, especially miRNAs, lncRNAs and circRNAs, play crucial roles in regulating AS. Recently, snRNAs (U1, U2, U4, U5, and U6) have also been shown to act as regulators of AS in cancer, in addition to basal factors of the spliceosome [41, 42]. In the following sections, we further discuss the specific regulatory relationships between ncRNAs and AS in cancers.

## Regulation of alternative splicing through cis-acting elements

Some endogenous ncRNAs that act as NATs (natural antisense transcripts), which are RNAs that contain sequences complementary to other endogenous RNAs, can specifically interact with cis-acting elements in pre-mRNAs by RNA–RNA base pairing. The interaction between ncRNAs and cis-acting elements can affect the selection of splice sites and/or the recruitment of SFs, thus regulating the expression of alternatively spliced isoforms in cancers. According to the origin of ncRNAs, their interaction with cis-acting elements has a cis-NAT form and a trans-NAT form.

**Fig. 3:**
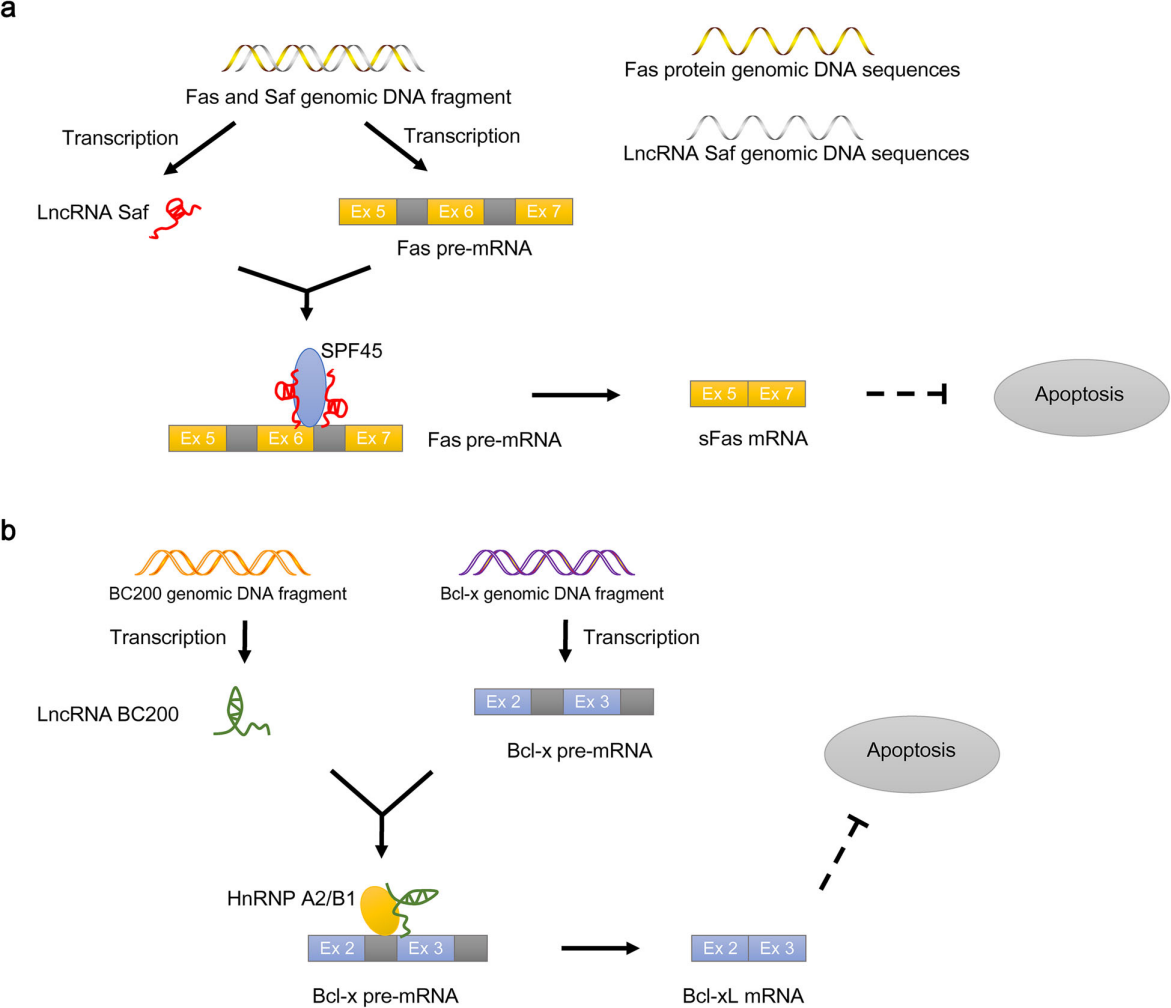
NcRNAs regulate AS through cis-acting elements. **a** One example of a cis-NAT: the lncRNA Saf binds specifically to the Ex 6 (exon 6) region of the Fas pre-mRNA and recruits SPF45, resulting in the expression of the sFas isoform, which consequently inhibits apoptosis. **b** One example of a trans-NAT: the lncRNA BC200 binds to Bcl-x pre-mRNA Ex 3 (exon 3) and recruits hnRNPA1/B2 to form the BC200-Bcl-x-hnRNPA2/B1 complex. This complex contributes to Bcl-xL expression and apoptosis resistance in breast tumor cells

A cis-NAT is transcribed from the opposite strand of a DNA strand at the same locus and usually has long perfect complementary sequences with the opposite transcript [43]. One example of a cis-NAT is the lncRNA Saf, which is transcribed from the opposite strand of the genomic DNA sequence of the Fas protein. Saf directly binds to the cis-acting element in the Fas pre-mRNA, and they form a specific double-stranded RNA intermediate located within or surrounded by the alternatively spliced exon 6, consequently recruiting the SF SPF45 to the Fas pre-mRNA. The interaction of Fas pre-mRNA, Saf, and SPF45 stimulates the exon skipping of Fas exon 6, resulting in the expression of the sFas (soluble Fas) isoform [44], which contributes to cancer survival by inhibiting Fas-mediated apoptosis in multiple cancers [45] (Fig. 3a). Another example of a cis-NAT is the lncRNA EGOT, which is transcribed from the opposite strand of the ITPR1 (inositol 1,4,5-trisphosphate receptor type 1) genomic sequence. EGOT can directly bind to the cis-acting element in ITPR1 pre-mRNA, resulting in the recruitment of the SF hnRNPH1 to promote hnRNPH1-mediated AS, contributing to the expression of the ITPR1 protein, which sensitizes cells to paclitaxel cytotoxicity in cancer therapy [46]. Additionally, the lncRNA *Zeb2* is a NAT and is transcribed from the opposite strand of the *Zeb2* protein genomic sequence, binds to the cis-acting element in *Zeb2* pre-mRNA and overlaps the splice site in the 5’UTR intron, preventing recognition of the spliceosome. The interaction between the *Zeb2* NAT and *Zeb2* pre-mRNA promotes the inclusion of the 5’UTR intron, thus increasing the levels of the Zeb2 protein, which can activate EMT (epithelial–mesenchymal transition) in breast cancer cells [47].

In contrast, ncRNAs can also interact with cis-acting elements through the trans-NAT form to regulate AS. A trans-NAT is transcribed from different genomic loci and usually has short and imperfect complementarity with the target pre-mRNA [43]. For example, the lncRNA BC200, derived from the BC200 gene, can directly bind the cis-acting element in the Bcl-x pre-mRNA derived from the Bcl-x gene [48, 49]. The lncRNA BC200 forms a specific double-stranded RNA intermediate with Bcl-x pre-mRNA exon 3, promoting the recruitment of the SF hnRNPA2/B1 to Bcl-x pre-mRNA. The interaction among BC200, hnRNPA2/B1 and Bcl-x pre-mRNA produces the Bcl-xL isoform and suppresses the expression of the Bcl-xS isoform [50], contributing to the apoptosis resistance of breast tumor cells [51] (Fig. 3b). Additionally, the lncRNA CCAT2, which is transcribed from genomic loci that is different from GLS (glutaminase), binds to an intron 14 fragment of GLS pre-mRNA and recruits the CFIm (cleavage factor I) complex to GLS pre-mRNA by direct binding. The CCAT2-CFIm-GLS interaction results in the expression of the GAC (glutaminase C) splicing isoform, promoting metastases and cell proliferation in CRC [52]. Moreover, the snRNA U1, which recognizes transcriptome-wide 5′ splice sites through base pairing [53], can bind to extensive U1-targeted pre-mRNAs to promote intron exclusion. Aberrant U1-mediated AS events generate many kinds of splicing isoforms and have a close association with characteristic breast cancer subtypes [41].

Therefore, some ncRNAs are able to interact with cis-acting elements through the cis-NAT or trans-NAT form, impacting AS outcomes in cancers.

## Regulation of alternative splicing through trans-acting factors

### Posttranscriptional regulation of splicing factors

NcRNAs have been observed as posttranscriptional regulators that participate in the regulation of the mRNA expression of SFs, thereby affecting splicing isoform outcomes.

**Fig. 4:**
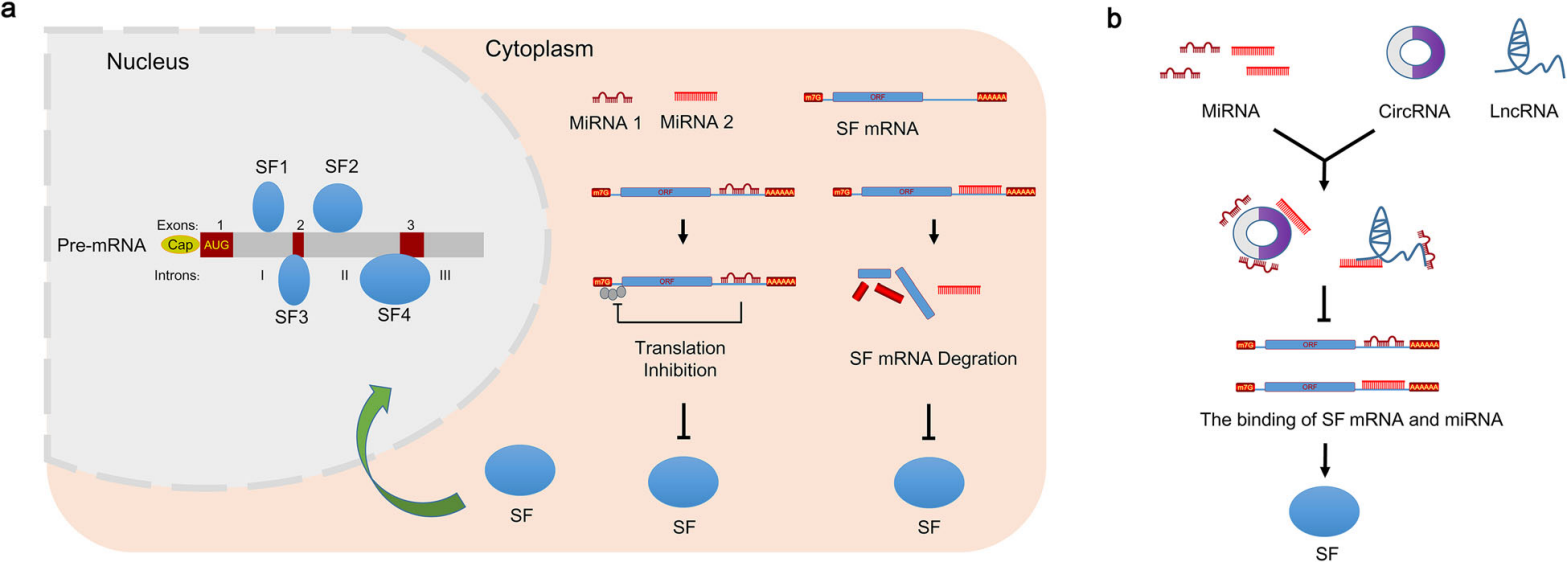
NcRNAs regulate AS through the posttranscriptional regulation of SFs. **a** MiRNAs silence SFs by directly binding to the SF mRNA. **b** CircRNAs and lncRNAs can sponge miRNA functions by directly binding to block their function with SF mRNAs, promoting SF expression

MiRNAs can inhibit SF expression through complete or partial complementation with the target mRNA of an SF, degradation of the mRNA or the downregulation of mRNA translation as a result [54, 55] (Fig. 4a). For example, in neuroblastoma, both miR-10a and miR-10b degrade SRSF1 mRNA through complete complementation with its 3’UTR and downregulate the translation of SRSF1 as a result of decreasing SRSF1-mediated AS, thus inhibiting the migration, invasion, and metastasis of neuroblastoma cells [56]. Additionally, miR-30c in PCa cells degrades SRSF1 mRNA through complete complementation with its 3’UTR and downregulates its translation to decrease SRSF1-mediated AS, thus suppressing PCa cell survival [57]. Moreover, in HCC (hepatocellular carcinoma), miRNA-133b inhibits the translation of SF3B4 (splicing factor 3b subunit 4) mRNA through partial complementation with its 3’UTR to decrease SF3B4-mediated AS, repressing cell proliferation and metastasis [58]. In HeLa cells, miR-7 inhibits the mRNA translation of the SF SRSF1 through partial complementation with its 3’UTR to downregulate SRSF1-mediated AS, suppressing cancer cell survival [59]. Here, we summarize the current miRNAs that regulate SFs at the posttranscriptional level Table 1.

**Table 1:** MiRNAs that regulate SFs at the posttranscriptional level

MiRNA	Splicing factor	Cancer	Reference
MiR-10a, miR-10b, miR-10b-5p, miR-203a-3p, miR30c	SRSF1	Neuroblastoma, renal cancer, prostate cancer	[56] [57], [60]
MiR-193a-3p, miR-200c-3p, miR-183-5p	SRSF2	Gastric cancer, renal cancer	[60], [61]
MiR-193a-5p	SRSF6	Pancreatic cancer	[61]
MiR-30a-5p, miR-216b-5p, miR-181a-5p, miR-7	SRSF7	Renal cancer, cervical cancer	[59], [62]
MiR-1	SRSF9	Bladder cancer	[63]
MiR-374b, miR-490, miR-149-5p, miR-135a-5p, miR-137	HnRNPA1	Hepatocellular carcinoma, gastric cancer, renal cancer	[64], [60], [65], [66]
MiR-124, miR-340	HnRNPA2	Colorectal cancer	[65]
MiR-335	Tra2β	Lung cancer	[67]
MiR-938	RBM5	Lung adenocarcinoma	[68]
MiR-335	RBM10	Endometrial cancer	[69]
MiR-137, miR-340, miR-133b, miR-124	PTBP1	Gastric cancer, colorectal cancer	[65], [70]
MiR-10b	MBNL1, MBNL2, DGCR14	Intracranial glioblastoma	[71]
MiR-200, miR-221, miR-375	QKI	Colorectal cancer, lung adenocarcinoma, oral squamous cell carcinoma, head and neck squamous cell carcinoma, breast cancer, prostate cancer	[72], [73], [74]
MiR-27a-3p	NOVA1	Gastric cancer	[75]
MiR-27b-3p	KHSRP	Breast cancer	[76]
MiR-184	SF1	Oral squamous cell carcinoma	[77]
MiR-361-3p, miR-615-5p	SF3B3	Non-small cell lung cancer	[78]
MiR-133b	SF3B4	Hepatocellular carcinoma	[58]

Some miRNAs and SFs form modulatory feedback circuits to maintain the coordination and relationship between miRNA-mediated SF gene repression and miRNA expression in AS regulatory networks. For instance, miR-7 prevents the translation of SRSF1 mRNA and decreases SRSF1-mediated AS, repressing cancer cell survival. In turn, SRSF1 directly binds to the long primary transcript of miR-7 called pri-miR-7, and SRSF1-independent splicing promotes pri-miR-7 processing into miR-7 by facilitating cleavage by Drosha [59]. Additionally, miR-181a-5p directly decreases both SRSF7 mRNA and protein levels, downregulating SRSF7-mediated AS, which reduces renal cancer cell proliferation. Conversely, SRSF7 contributes to miR-181a-5p expression through a possible mechanism that directly facilitates maturation of the miR-181a-5 primary transcript [62].

In addition, circRNAs and lncRNAs have been observed to promote SF expression by binding and sponging miRNAs as ceRNAs (competing endogenous RNAs), thus regulating AS in cancers (Fig. 4b). For example, in non-small cell lung cancer, the circRNA 100,146 can bind and sponge miR-361-3p and miR-615-5p, which both directly inhibit the expression of the SF SF3B3, thus increasing SF3B3-mediated AS to promote the proliferation and invasion of cancer cells [78]. Similarly, the lncRNA UCA1 can bind and sponge miR-184, which directly decreases the expression of SF1, resulting in the upregulation of SF1-mediated AS to accelerate the proliferation and cisplatin resistance of oral squamous cell carcinoma [77]. The lncRNA CCAT1 binds and sponges miR-490, which directly inhibits the expression of the SF hnRNPA1, consequently increasing hnRNPA1-mediated AS to promote gastric cancer cell migration [64].

### Posttranslational regulation of splicing factors

Acting as posttranslational regulators of SFs, ncRNAs can control the posttranslational chemical modifications (e.g., phosphorylation) of SFs or decoy SFs, remodel chromatin, or encode peptides to interact with SFs, thus altering splicing outcomes in cancers.

#### Posttranslational chemical modifications

**Fig. 5:**
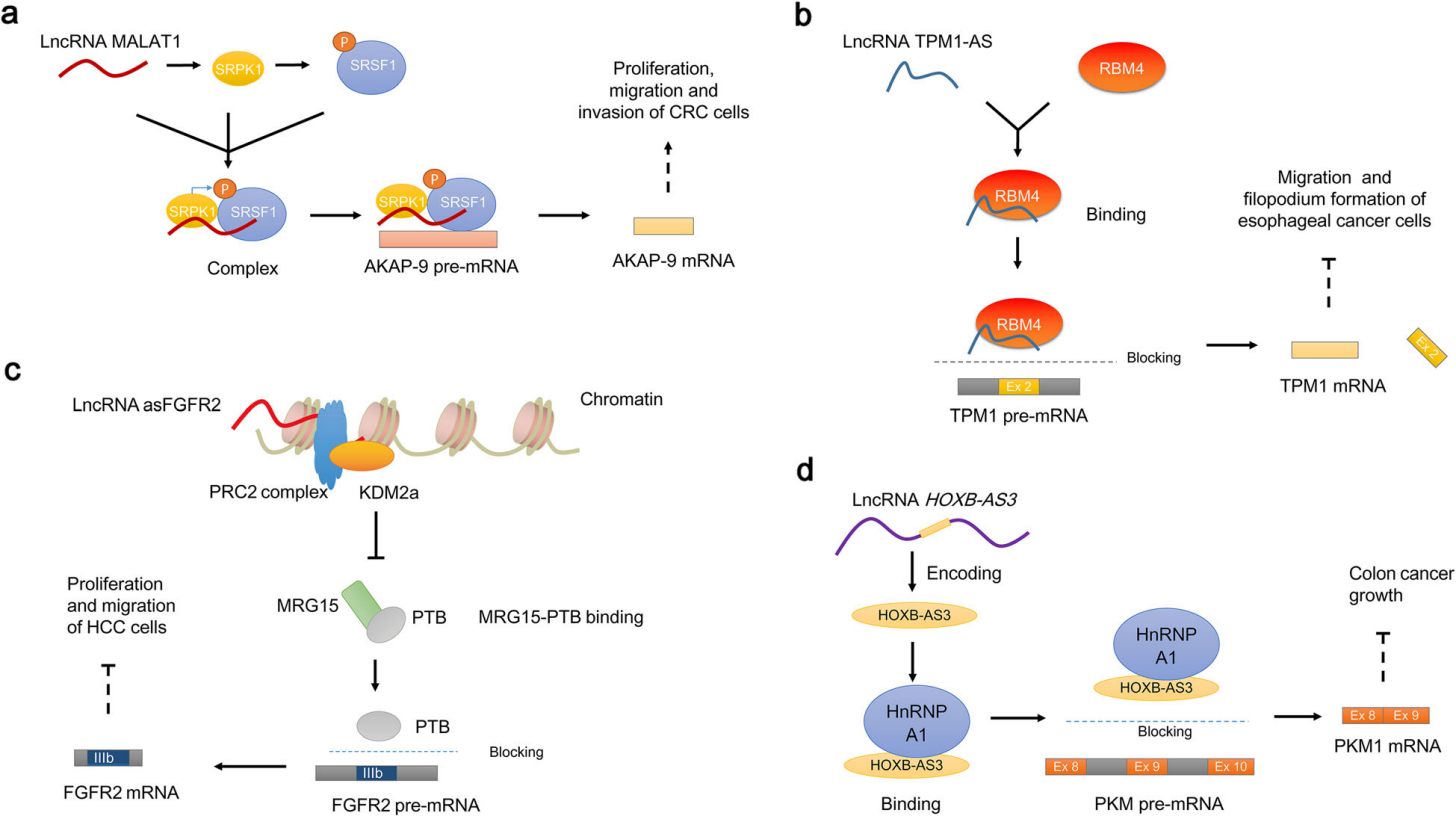
NcRNAs regulate AS through the posttranslational regulation of SFs. **a** Posttranslational chemical modifications. The lncRNA MALAT1 contributes to SRSF1 phosphorylation by stimulating both the expression and activity of SRPK1, thereby promoting SRSF1-mediated AS of the AKAP-9 pre-mRNA and enhancing the expression of the AKAP-9 isoform, which exacerbates CRC. **b** Decoy SFs. For example, the lncRNA TPM1-AS can directly block the binding between the SF RBM4 and TPM1 pre-mRNA and regulate AS, inhibiting the progression of cancer. **c** Chromatin remodeling. The lncRNA asFGFR2 recruits PRC2 and KDM2a to chromatin to form a chromatin environment that disrupts the binding of MRG15-PTB. This disruption alters the location of the SF PTB, promoting FGFR2 mRNA with exon IIIb inclusion and consequently repressing HCC progression. **d** Encoding functional peptides. The lncRNA *HOXB-AS3* encodes the peptide HOXB-AS3, which targets hnRNPA1 and blocks it from binding to PKM pre-mRNA flanking exon 9, decreasing the expression of the PKM2 isoform and consequently repressing the growth of colon cancer

NcRNAs are able to control SF phosphorylation, which enhances SF activity to promote AS. In CRC cells, the lncRNA MALAT1 has been found to form a complex with both SRSF1 and SRPK1 (serine/arginine protein kinase 1) in nuclear speckles [79]. MALAT1 can increase both the expression and activity of SRPK1 [80], which functions to catalyze SRSF1 phosphorylation by binding to the RS domain of unphosphorylated SRSF1 [81]. MALAT1-mediated SRSF1 phosphorylation can affect the subcellular localization of SRSF1 and promote splice site selection in AKAP-9 (A-kinase anchor protein 9) pre-mRNA [82–84], thus contributing to the expression of the AKAP-9 isoform, which exacerbates cellular proliferation, migration and invasion in CRC [80] (Fig. 5a).

#### Decoy splicing factors

Some ncRNAs can act as “decoys” to inhibit SFs binding to targeted pre-mRNAs through direct interactions, thus regulating AS in cancers [85–87]. For example, the lncRNA TPM1-AS binds to the SF RBM4 and blocks the targeting of TPM1 pre-mRNA, leading to the inhibiting of TPM1 exon 2a inclusion and a decrease in the two tumorigenic splicing isoforms V2 and V7 and thus inhibiting migration and filopodium formation in esophageal cancer cells [88] (Fig. 5b). Additionally, the lncRNA PNCTR can recruit at least 6 of the SF PTBP1 (polypyrimidine tract binding protein 1) molecules and sequester them in a nuclear body called the PNC (perinucleolar compartment), hindering PTBP1 from acting on CHEK2 (checkpoint kinase 2) pre-mRNA and contributing to the expression of a CHEK2 isoform that includes exon 8. The lncRNA PNCTR-mediated splicing isoform promotes cell survival in many kinds of cancers [89]. Moreover, the circRNA circSMARCA5 binds and decoys the SF SRSF1 away from VEGF-Axxxa pre-mRNA to decrease the expression of the proangiogenic splicing isoform VEGF-Axxxa, repressing cancer progression in glioblastoma multiforme [90]. Additionally, the circRNA circ-UBR5 can recruit the SFs QKI and NOVA1 and block their splicing functions to change the AS outcome, affecting tumor differentiation in non-small cell lung cancer [91]. It should be noted that a series of studies on the decoy roles by which ncRNAs inhibit SF-mediated AS in cancers have been conducted Table 2. In addition to the inhibitory function described above, ncRNAs can also act as “decoys” to promote the binding of SFs to targeted pre-mRNAs. For example, the lncRNA PCGEM1 can bind and enhance the binding capacity of the SF U2AF65 to AR pre-mRNA and promote the expression of the AR3 splicing isoform by exonization, contributing to the development of PCa [87, 100].

**Table 2:** NcRNAs as SF decoys to inhibit SF-mediated AS in cancer

NcRNA	Splicing factor	Cancer	Reference
CircSMARCA5	SRSF1	Glioblastoma multiforme	[90]
LINC01133	SRSF6	Colorectal cancer	[92]
PCGEM1	HnRNPA1	Prostate cancer	[87]
TPM1-AS	RBM4	Esophageal cancer	[88]
FAS-AS1	RBM5	B-cell lymphomas	[93]
PNCTR, PANDAR, LUCAT1	PTBP1	Cervical cancer, osteosarcoma, colorectal cancer	[89], [94] [95]
circ-UBR5	QKI, NOVA1	Non-small cell lung cancer	[91]
MALAT1	SFPQ	Colorectal cancer	[96]
Gomafu	SF1, Celf3	Neuroblastoma	[97]
Spry1	U2AF65	Breast cancer	[98]
*LASTR*	SART3	Breast cancer	[99]

#### Chromatin remodeling

NcRNAs can fine-tune the chromatin structure, which controls the recruitment of SFs to affect their interaction with pre-mRNAs in the AS process, changing splicing outcomes. The lncRNA asFGFR2 has been verified to function through the recruitment of PRC2 (polycomb repressive complex 2, one of the two classes of polycomb-group proteins) and the histone demethylase KDM2a (lysine-specific demethylase 2A) to chromatin to form a special chromatin environment that disrupts the binding between MRG15 (MORF4-related gene on chromosome 15) with PTB (polypyrimidine tract binding protein). The disruption of the MRG15-PTB interaction alters the location of the SF PTB and decreases the recruitment of the SF PTB to exon IIIb, promoting exon IIIb inclusion in the course of FGFR2 (fibroblast growth factor receptor 2) splicing [101] and consequently producing the FGFR2-IIIb isoform, which decreases the proliferation and migratory potential of HCC [102] (Fig. 5c).

#### Encoding functional peptides

NcRNAs, especially lncRNAs, are able to regulate SFs at the posttranslational level through limited coding potential. For example, the lncRNA *HOXB-AS3* encodes a conserved HOXB-AS3 peptide, and the HOXB-AS3 peptide competitively targets arginine residues in an RNA-binding area (called an RGG box) of hnRNPA1, which blocks the binding of hnRNPA1 to certain sequences of the PKM (pyruvate kinase M) pre-mRNA flanking exon 9, thereby inhibiting hnRNPA1-mediated AS, decreasing the expression of the PKM2 isoform and consequently repressing the growth of colon cancer [103] (Fig. 5d).

## Regulation of alternative splicing through pre-mRNA transcription

AS is not an independent posttranscriptional regulation but is intimately coupled with transcription, and factors that regulate pre-mRNA transcription can have an impact on AS [10]. Emerging roles of ncRNAs have been found to directly participate in transcription [31, 104, 105] and may therefore impact the AS outcome. For example, the lncRNA EZR-AS1 can form a complex with Pol II and recruit the histone H3-lysine 4-specific methyltransferase SMYD3 (SET and MYND domain containing protein 3) to a site downstream of the transcription promoter. The interaction of EZR-AS1 with Pol II and SMYD3 promotes the histone modification H3K4me3 (trimethylation of lysine 4 on histone H3) in transcription sites and facilitates pre-mRNA transcription [106, 107]. H3K4me3 is also able to recruit spliceosome components to promote the exclusion of exon 2 from the MCL1 (myeloid cell leukemia 1) pre-mRNA, resulting in the expression of the MCL1S isoform, which induces cancer cell apoptosis [107–110]. In addition, the lncRNA HOTAIR decreases the recruitment of multiple promoting molecules of SETD2 (SET domain-containing 2, the specific methyltransferase of H3K36) to downregulate SETD2 expression and phosphorylation, thus reducing SETD2-mediated H3K36me3, which can activate pre-mRNA transcription [111, 112]. The reduction in H3K36me3 promotes the expression of the CDH1 (cadherin 1) isoform with exon 8, acting as a biological marker in gastric cancer occurrence [113]. Furthermore, the lncRNA DACOR1 in colon cancer cells promotes genome-wide DNA methylation [114, 115]. Interestingly, not correlating with the negative regulation of transcription, DNA methylation at Oct4A gene enhancers can facilitate the expression of the Oct4A splicing isoform by affecting the AS of Oct4A pre-mRNA, inhibiting cell aging in embryonal carcinoma [115, 116].

In conclusion, ncRNAs may regulate AS by affecting transcription, mainly by interacting with Pol II and regulating histone modification and DNA methylation.

### Diagnostic and therapeutic value of ncRNA-regulated alternative splicing in cancer

High-throughput sequencing technology has allowed the measurement of an AS event in terms of an overall ratio, and the final results make it possible to provide diagnostic information in this manner. An increasing number of studies have indicated that ncRNAs can serve as biomarkers for diagnosis or drug resistance in many types of cancers due to their participation in AS events [61, 64, 117–121]. For example, the lncRNA HOXA11-AS can regulate SF SRSF1-mediated AS, contributing to GC (gastric cancer) cell proliferation and invasion. Expression of the lncRNA HOXA11-AS was found to be increased in GC tissues and serum samples. Clinical data indicate that GC patients with a high HOXA11-AS level have a poor prognosis and that HOXA11-AS may function as a potential diagnostic biomarker in GC [117]. Additionally, the lncRNA 91H has been verified to regulate hnRNPK-mediated AS, affecting cancer processes such as metastasis in CRC. Expression of the lncRNA 91H in the serum was found to be increased in CRC patients and is considered an early clinical marker for CRC recurrence and metastasis [119]. Additionally, the lncRNA LINC01133 can block the splicing function of SRSF6 [92], which contributes to the EMT process by promoting the expression of the tumorigenic splicing isoform [22]. Analyses of clinical CRC tissues have shown that patients with high LINC01133 expression always have better survival outcomes than those with low expression and that LINC01133 may act as a prognostic biomarker due to its antimetastatic function. In addition, miR-193a-3p can downregulate AS events mediated by the SF SRSF2, thereby contributing to cisplatin resistance. Analyses of clinical GC tissues have suggested that some patients with high miR-193a-3p expression may have a high level of resistance to cisplatin and that miR-193a-3p may act as a clinical biomarker of cisplatin resistance in GC patients after surgery [122]. Moreover, the lncRNA EGOT participates in the AS of ITPR1 pre-mRNA, sensitizing cancer cells to paclitaxel treatment. Analyses of clinical breast cancer samples have shown that patients with low EGOT expression have a high level of paclitaxel resistance and that EGOT may be utilized as a diagnostic biomarker of paclitaxel treatment for breast cancer patients after surgery [46].

**Fig. 6:**
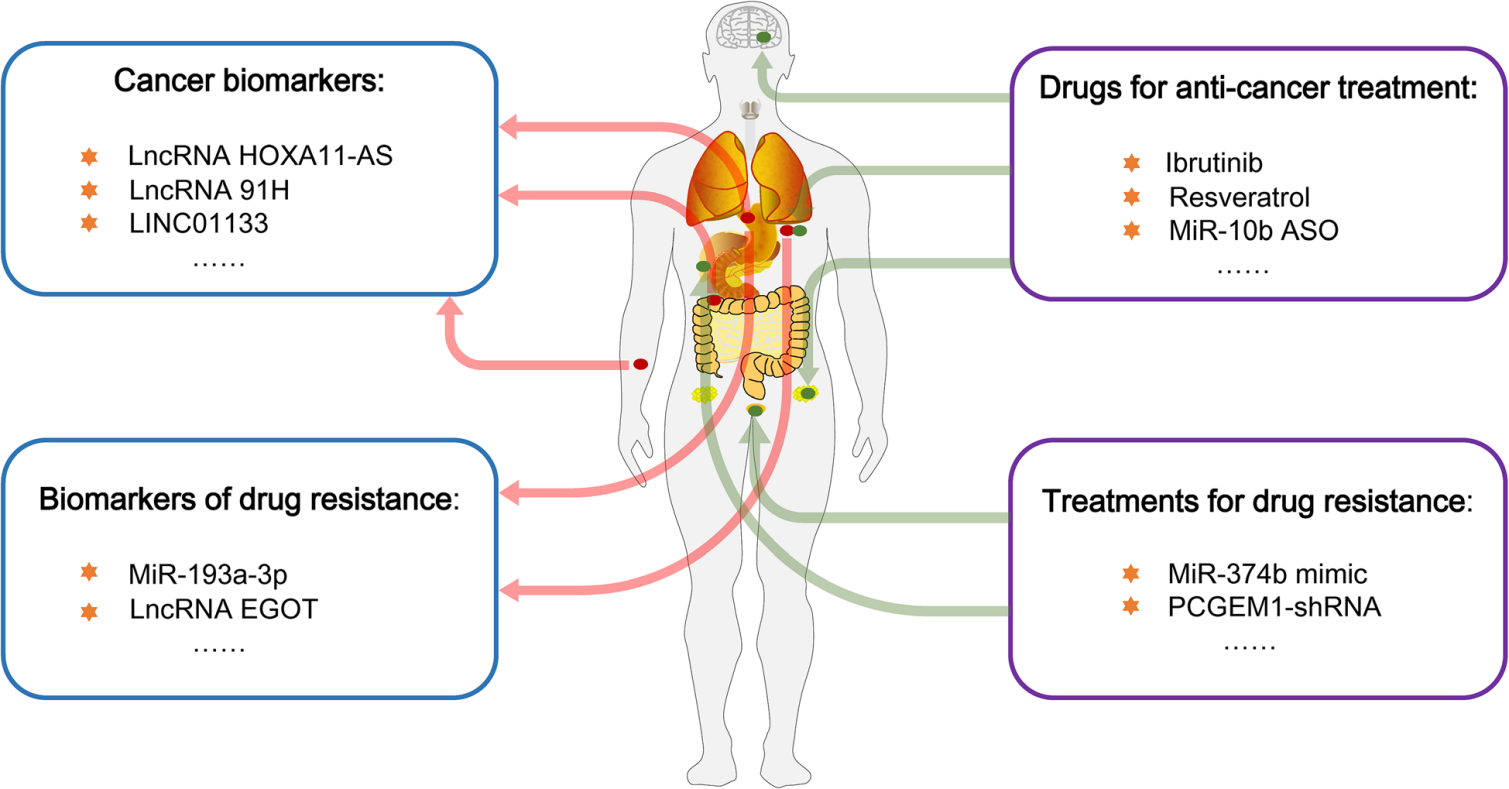
A schematic representation: the diagnostic and therapeutic value of ncRNA-regulated AS in cancer. Multiple ncRNAs can act as diagnostic biomarkers and promising targets for pharmaceutical development in various types of cancers due to their participation in AS events

In view of the important regulatory roles in AS, ncRNAs have rapidly emerged as promising targets for pharmaceutical development in cancers [67, 68, 92, 123–131]. For example, the lncRNA FAS-AS1 promotes the expression of the splicing isoform sFas by directly binding with the SF RBM5, thus increasing resistance to apoptosis in B-cell lymphomas [93]. Ibrutinib has been proven to repress the splicing isoform mediated by RBM5 for cell-killing effects by downregulating FAS-AS1 expression, and it has been applied to target B-cell malignancies as an anticancer drug [93, 132, 133]. Additionally, miR-424 and miR-503 can regulate AS events mediated by the SF hnRNPA1, decreasing breast cancer cell proliferation [134]. Resveratrol, a polyphenolic phytoalexin with multiple functions, such as antitumorigenic and anti-inflammatory effects [135, 136], is a popular health care product on the market. It has been found that resveratrol is able to promote the expression of the tumor-suppressive miRNAs miR-424 and miR-503, and it has been regarded as a promising application for cancer prevention and treatment [134]. Furthermore, with high expression in all GBM (glioblastoma) subtypes, oncogenic miR-10b possesses strong therapeutic potential due to its regulatory function in AS by interacting with multiple SFs, including MBNL1 (muscleblind-like splicing regulator 1), MBNL2 and DGCR14 (DiGeorge syndrome critical region gene 14) [71, 137]. Various formulations of miR-10b ASO (antisense oligonucleotide) inhibitors delayed GBM progression in mouse models through three routes (direct intratumoral injection, continuous osmotic delivery, and systemic intravenous injection) without significant systemic toxicity, indicating that a miR-10b inhibitor can be used as a drug against all GBM subtypes [71]. Moreover, in HCC, miR-374b can play an AS regulatory role in the miR-374b/hnRNPA1/PKM2 axis, addressing the problem of sorafenib resistance. MiR-374b mimic was transfected by lipofectamine, which was injected into SCID mice sensitized HCC cells to sorafenib toxicity, indicating that miR-374b is an effective treatment for sorafenib resistance [66]. In addition, in prostate cancer, PCGEM1 inhibits HnRNPA1-mediated AS in androgen receptor pre-mRNA, promoting castration resistance (including drug resistance to enzalutamide and abiraterone) [87, 138]. PCGEM1-shRNA, through lentiviral packaging and injection into castrated male SCID mice, restrains tumor growth, suggesting that PCGEM1 can act as a likely drug target of castration resistance [87] (Fig. 6).

## Conclusions

We summarize the recent ncRNAs, mainly miRNAs (Table 1) and lncRNAs (Table 3), that regulate AS at multiple levels in cancer. Notably, current related observations focus mainly on regulation through cis-acting elements or trans-acting factors based on the posttranscription of nascent pre-mRNAs. Considering that a pre-mRNA indicates simply an immature and temporary state (as a presumptive primary transcript) and that the process of transcription is accompanied by splicing, ncRNA-regulated transcription could have a comprehensive and complex impact on AS, including the maturation and selection of cis-acting elements and the recruitment of trans-acting factors. The direct observation of the functions of ncRNAs in transcription and AS is a potential research direction in the AS domain of cancers.

**Table 3:** LncRNAs that regulate AS in cancer

LncRNA	Function	Cancer	Reference
Saf	Interaction with Fas pre-mRNA through the cis-NAT form	Leukemia, cervical cancer	[44]
EGOT	Interaction with ITPR1 pre-mRNA through the cis-NAT form	Breast cancer	[46]
*Zeb2*	Interaction with *Zeb2* pre-mRNA through the cis-NAT form	Breast cancer	[47]
BC200	Interaction with Bcl-x pre-mRNA through the trans-NAT form	Breast cancer	[50]
CCAT2	Interaction with GLS pre-mRNA through the trans-NAT form	Colorectal cancer	[52]
UCA1	Sponging miR-184-mediated AS	Oral squamous cell carcinoma	[77]
CCAT1	Sponging miR-490-mediated AS	Gastric cancer	[64]
MALAT1	Promoting SRPK1-catalyzed SRSF1 phosphorylation	Colorectal cancer	[80]
LINC01133	Decoying the SF SRSF6 away from the targeted pre-mRNA	Colorectal cancer	[92]
PCGEM1	Decoying the SF HnRNPA1 away from the targeted pre-mRNA	Prostate cancer	[87]
TPM1-AS	Decoying the SF RBM4 away from the targeted pre-mRNA	Esophageal cancer	[88]
FAS-AS1	Decoying the SF RBM5 away from the targeted pre-mRNA	B-cell lymphomas	[93]
PNCTR, PANDAR, LUCAT1	Decoying the SF PTBP1 away from the targeted pre-mRNA	Cervical cancer osteosarcoma, colorectal cancer	[89], [94], [95]
MALAT1	Decoying the SF SFPQ away from the targeted pre-mRNA	Colorectal cancer	[96]
Gomafu	Decoying the SF SF1 and Celf3 away from the targeted pre-mRNA	Neuroblastoma	[97]
Spry1	Decoying the SF U2AF65 away from the targeted pre-mRNA	Breast cancer	[98]
*LASTR*	Decoying the SF SART3 away from the targeted pre-mRNA	Breast cancer	[99]
asFGFR2	Fine-tuning the chromatin structure to decrease the recruitment of the SF PTB	Breast cancer	[101]
HOXB-AS3	Encoding peptides to inhibit hnRNPA1-mediated AS	Colon cancer	[103]

The number of current studies on the association of ncRNAs with the regulation of AS in cancer is limited. In view of the use of new technologies represented by high-throughput sequencing and further experiments with innovative designs, it is predictable that many AS-associated ncRNAs, splicing isoforms and new regulatory mechanisms of cancer progression will be found and may provide potential targets for cancer diagnosis and therapy. Additionally, questions remain in the field of small molecules against ncRNA targets. One key consideration is how to classify and evaluate ncRNA targets, as ncRNA targets usually do not have “active sites” [139]. In addition, ncRNA therapeutics may need solutions for drug delivery, toxic side effects, and drug resistance [140, 141].

## Data Availability

All data generated or analyzed during this study are included in this published article.

## Abbreviations

AS: Alternative splicing
ncRNA: Noncoding RNA
pre-mRNA: Pre-messenger RNA
snRNPs: Small nuclear ribonucleoprotein
ISE: Intron splicing enhancer
ISS: Intron splicing silencer
ESE: Exon splicing enhancer
ESS: Exon splicing silencer
ES: Exon skipping
IR: Intron retention
MXE: Mutually exclusive exon
A5SS: Alternative 5′ splice site
A3SS: Alternative 3′ splice site
SF: Splicing factor
SR: Serine/arginine-rich
hnRNP: Ribonucleoprotein
RBM5: RNA binding motif protein 5
RBM24: RNA binding motif protein 24
QKI: Quaking
Nova1: NOVA alternative splicing regulator 1
SRSF1–12: Serine/arginine-rich splicing factor 1–12
U2AF: U2 auxiliary factor
SF3B3: Splicing factor 3b subunit 3
Pol II: RNA polymerase II
H3K36me3: Trimethylation on histone H3 thirty-sixth lysine
CRC: Colorectal cancer
PCa: Prostate cancer
TNC: Tenascin C
miRNA: MicroRNA
siRNA: Small interfering RNA
piRNA: Piwi-interacting RNA
rRNA: Ribosomal RNA
snRNA: Small nuclear RNA
lncRNA: Long noncoding RNA
circRNA: Circular RNA
RISC: RNA-induced silencing complex
UTR: Untranslated region
NAT: Natural antisense transcript
sFas: Soluble Fas
ITPR1: Inositol 1,4,5-trisphosphate receptor type 1
EMT: Epithelial–mesenchymal transition
GLS: Glutaminase
CFIm: Cleavage factor I
GAC: Glutaminase C
Ex: Exon
HCC: Hepatocellular carcinoma
SF3B4: Splicing factor 3b subunit 4
ceRNA: Competing endogenous RNA
SRPK1: Serine/arginine protein kinase 1
AKAP-9: A-kinase anchor protein 9
PTBP1: Polypyrimidine tract binding protein 1
PNC: Perinucleolar compartment
CHEK2: Checkpoint kinase 2
PRC2: Polycomb repressive complex 2
KDM2a: Lysine-specific demethylase 2A
MRG15: MORF4-related gene on chromosome 15
PTB: Polypyrimidine tract binding protein
FGFR2: Fibroblast growth factor receptor 2
PKM: Pyruvate kinase M
SMYD3: SET and MYND domain containing protein 3
H3K4me3: Trimethylation of lysine 4 on histone H3
MCL1: Myeloid cell leukemia 1
SETD2: SET domain-containing 2
CDH1: Cadherin 1
GC: Gastric cancer
GBM: Glioblastoma
MBNL1: Muscleblind like splicing regulator 1
DGCR14: DiGeorge syndrome critical region gene 14
ASO: Antisense oligonucleotide
